# Pilot evaluation on an adapted tele-behavioral activation to increase physical activity in persons with depression: a single-arm pilot study

**DOI:** 10.1186/s40359-024-02053-5

**Published:** 2024-11-09

**Authors:** Chad D. Rethorst, Joseph M. Trombello, Patricia M. Chen, Thomas J. Carmody, Lynnel C. Goodman, Alejandra Lazalde, Madhukar H. Trivedi

**Affiliations:** 1 Institute for Advancing Health Through Agriculture, Texas A&M University System, Dallas, TX USA; 2 Department of Psychiatry, University of Texas Southwestern Medical Center, Dallas, TX USA; 3 O’Donnell School of Public Health, University of Texas Southwestern Medical Center, Dallas, TX USA; 4 Department of Population and Data Sciences, University of Texas Southwestern Medical Center, Dallas, TX USA; 5 Ellie Mental Health, Dallas, TX USA; 6 Texas A&M Agrilife Research and Extension Center – Dallas, 17360 Coit Rd, Dallas, TX 75252 USA

**Keywords:** Depression, Behavioral activation, Physical activity, Exercise

## Abstract

**Background:**

Physical activity has the potential to improve physical and mental health outcomes of persons with depression. However, feasible and acceptable strategies to integrate physical activity interventions into real-world settings are needed.

**Objective:**

To assess the feasibility and acceptability of a manualized Behavioral Activation intervention aimed to increase physical activity in persons with depression (defined as a PHQ-9 score *≥* 10).

**Methods:**

A single-arm pilot study was conducted. The intervention consisted of 8 tele-therapy sessions delivered over a 10-week period. Measures of feasibility included screening, enrollment, intervention adherence, outcome data availability, and intervention fidelity. Acceptability was assessed with a post-intervention survey and qualitatively through focus groups and interviews. Preliminary efficacy of the intervention was assessed by evaluating pre-to-post changes in physical activity and depressive symptoms.

**Results:**

All feasibility metrics exceeded predetermined feasibility goal metrics with the exception of Fitbit wear and screening rate, which was due to a greater than anticipated enrollment rate. Participants (*n* = 15) reported perceived benefits from the intervention and convenience in attending tele-therapy sessions. Depressive symptoms, as measured by the PHQ-9 improved (16.8 at enrollment to 10.1 post intervention, Cohen’s *d = 1.13*). Self-reported moderate-to-vigorous physical activity (MVPA) increased from 22.0 min/week at baseline to 36.67 min/week post-intervention (*d* = 0.58). Physical activity as measured by the Fitbit showed little change (daily step 5543.29 during Week 1 to 6177.48 during Week 10, (*d* = 0.14); MVPA 21.23 min/week during Week 1 to 19.22 at Week 10 (*d* = 0.0.06).

**Conclusions:**

Results of the pilot study suggest the intervention is feasible to deliver and acceptable to participants. Preliminary results suggest the intervention may be effective in improving depressive symptoms and increasing self-reported physical activity.

**Trial registration:**

ClinicalTrials.gov NCT04990401, Registered July 21, 2021.

**Supplementary Information:**

The online version contains supplementary material available at 10.1186/s40359-024-02053-5.

## Background

Persons with depression experience significant disease burden [1, 2], including increases in mortality and morbidity. In a recent meta-analysis, persons with Major Depressive Disorder had a 58% increase in mortality risk compared to those without depression, while the increased risk among those with subthreshold depressive symptoms was 33% [3]. It is estimated that only one-third of patients achieve remission following initial treatment, and even following multiple treatment attempts, one-third of patients fail to achieve remission [4–6]. Among those who do achieve remission during treatment, 85% will experience a recurrence within a 15-year period [7]. Depression is also associated with an increased risk of developing other chronic health conditions, including cardiovascular disease, type 2 diabetes, and cancer. The comorbidity between depression and chronic physical health conditions may be related to general behavioral avoidance, including of health-promoting behaviors like healthy eating and physical activity [8–12]. The above factors indicate the need for improved treatment options to improve depressive symptoms and reduce depression relapse and highlight the need for treatments that can also reduce the future risk of developing other chronic diseases.

Physical activity, including structured exercise interventions, has the potential to improve treatment outcomes and long-term health outcomes of persons with depression. Meta-analyses of randomized controlled trials indicate that the treatment effect of exercise interventions is comparable to that of psychotherapy and antidepressant medication as a stand-alone treatment [13–15], and has proven efficacious as an adjunctive or augmentation treatment along with other treatments [16, 17]. While follow-up data from these trials is limited, data from a one-year follow-up in one trial suggests patients who engaged in regular physical activity are less likely to be depressed [18]. The general health benefits of physical activity are well documented [19]. Specifically in the context of depression, data indicates that higher cardiovascular fitness reduces the morality risk for persons with a history of depression [20]. 

Despite the potential benefits of exercise interventions, several barriers exist that limit the use of structured exercise interventions or interventions to increase physical activity among persons with depression in clinical settings. Physicians often note they lack training in exercise prescription due to an educational focus on pharmaceuticals [21] and lack knowledge regarding the role of exercise physiologists within the medical settings [22]. As a result, clinicians often prefer to send patients to resources outside the clinical setting for physical activity support [21–23]. In contrast to many countries that provide support within the medical system for exercise or physical activity support, cost-reimbursement within the United States for exercise and physical activity is also limited [24], further inhibiting the use of such programs in clinical settings. As such exercise is rarely used as a treatment for depression by clinicians; in a survey of general practitioners in the United Kingdom, only 5% of practitioners include exercise in their top three treatment choices [25]. 

In response to these barriers, we developed an adapted Behavioral Activation (BA) intervention focused on increasing physical activity and decreasing depressive symptoms [26]. This approach aims to address limitations in previous interventions, including challenges in participant adherence and also in maintenance once research-based interventions end. In prior studies evaluating a combination of BA and exercise, adherence was 50% or lower [27–30]. These interventions were also 16 weeks or longer in duration, and required in-person attendance of a significant number of intervention sessions. These factors are likely to contribute to poor adherence, maintenance of physical activity post-intervention, and incorporation of physical activity into daily life. In contrast, our BA intervention will be of a limited duration (8 sessions) and delivered through teletherapy in an aim to ameliorate logistical barriers to recruitment, study retention, and intervention adherence. Our team has previously demonstrated that manualized BA delivered via teletherapy to persons with depression results in reductions in depression and anxiety symptoms [31, 32]. In this paper we report the results of Phase 1 of our project (ClinicalTrials.gov NCT04990401) evaluating this approach. The aim of this phase was to conduct a mixed-methods pilot study to assess the feasibility and acceptability of the manualized BA intervention. A full description of the study procedures has been previously published [26]. 

## Methods

### Overview

The pilot study was a single-arm design. Individuals completed online and in-person screening to determine eligibility. Participants completed a baseline data collection visit and attended 8 intervention sessions delivered over a 10-week period, followed by 2 bi-weekly “booster” sessions. Given the flexibility in intervention session scheduling, final data collection occurred between Weeks 14–16.

### Participants

We recruited participants from the Center for Depression Research and Clinical Care at UT Southwestern Medical Center, specifically through an ongoing Dallas 2 K study, a longitudinal study of children, adolescents, adults and elderly adults with a current or prior history of unipolar depression, and the Mood Disorders Network, a network of primary and specialty care clinics, including charity clinics, with connections to the Center for Depression Research and Clinical Care. We included individuals ages 18 to 64 who had moderate to severe depressive symptoms, defined as a PHQ-9 [33] score *≥* 10, engaged in less than 90 min of moderate-to-vigorous physical activity (MVPA) per week as measured by the Physical Activity Vital Sign (PAVS) [34], and had a smartphone to use for teletherapy sessions. Individuals with current, past, or lifetime manic, or hypomanic episode, psychosis, schizophrenia or schizophreniform disorder, currently experiencing suicidal ideation with plan and intent or in current, active psychotherapy, or had a medical condition prohibiting physical activity were excluded. To determine eligibility, individuals completed a pre-screen questionnaire and in-person screening visit, led by a member of the research team with training in diagnostic interviews, and were subsequently enrolled in the study. Participants completed in-person screening visits between August and December 2021 at the Center for Depression Research and Clinical Care. This study was approved by the Institutional Review Board at UT Southwestern Medical Center (IRB # STU-2021-0441) and all participants signed an informed consent prior to participation.

### Intervention

The manualized intervention consisted of 10 BA individual teletherapy sessions; eight weekly BA teletherapy sessions, which were to be completed within a 10-week period to allow for flexibility in scheduling, followed by two biweekly booster sessions (delivered between Weeks 10–14). Teletherapy was delivered virtually, through either live video-based conferencing integrated in with the electronic medical record system or through telephone visits. The BA was delivered by a licensed masters-level mental health professional, typically licensed in social work or professional counseling. The treatment manual was adapted from a BA manual that demonstrated efficacy for reducing depressive symptoms [31, 32], and was adapted to include specific recommendations and support for increasing physical activity to help participants schedule and engage in physical activity and problem-solve potential barriers to physical activity. Particapnts were asked to schedule at least 2 activities per week related to physical activity and tracked their activity in a BA workbook, although additional activities unrelated to physical activity but associated with pleasure and/or mastery/accomplishment could also be executed.

### Data collection

#### Patient health questionnaire (PHQ-9)

The PHQ-9 is a self-report measure of depressive symptoms aligned with the DSM-5 criteria for depression [33]. Participants completed the PHQ-9 prior to each intervention session during the intervention period and at follow-up.

#### Physical activity

Participants were asked to wear a Fitbit Inspire HR™ throughout their participation, removing it only for charging. The Fitbit provides minute-level data and wear-time was determined by the presence of a heartrate value at each minute. Times of sleep were identified using the Fitbit generated sleep score metric that was also determined on the minute-level. Valid wear days was defined as days with a non-sleep wear time of at least 600 min. “Active Minutes” captured by the Fitbits have demonstrated reliability and validity compared to moderate-to-vigorous activity measured by Actigraph [35]. Self-reported physical activity was measured using the Physical Activity Vital Sign (PAVS), a 2-item assessment with demonstrated reliability and validity [34, 36, 37], that was completed prior to each intervention session and at follow-up.

#### Intervention fidelity

The Quality of Behavioral Activation Scale - Short Form (QBAS- SF) is a 14-item scale used to rate the treatment fidelity of BA clinicians, with an overall average score of 3 or higher (range: 0–6) per item as the cutoff for intervention fidelity [38, 39]. Session were recorded and approximately 20% of sessions were randomly selected and scored for fidelity by a co-investigator.

### Satisfaction/acceptability

#### Post-initial session questionnaire

Following the initial intake session, participants completed a brief 7-item questionnaire (see *Supplementary Materials)*, created for this study, to assess their satisfaction with the visit, their intention to continue in the intervention, and potential barriers and facilitators to participation.

#### Exit survey upon intervention completion

Participants completed a 21-item exit survey, created for this study (see *Supplementary Materials)*, at the completion of the intervention to assess intervention acceptability, the perceived benefits of the usefulness of the intervention, and the likelihood that patients would choose such an intervention in the future.

#### Focus groups

Two focus groups were conducted by PC with participants who completed the intervention (“completers”). Each focus group consisted of five individuals and lasted approximately 60 min. Focus group topics explored participants’ experience with the intervention, perceptions of its impact on their physical activity and depressive symptoms, and suggestions on how to improve the intervention. Focus groups were audio-recorded and transcribed for analysis. The groups were semi-structured, driven by a focus group guide but with flexibility to engage in discussion on related topics. The guides gathered data on participants’ experiences and opinions of the intervention, the intensity of completed exercise, barriers and facilitators of intervention adherence, and perceived benefits of the intervention. We also elicited participants’ receptiveness to the intervention, learned how the intervention influenced their adherence to physical activity, and gauged their opinions on the teletherapy format.

#### Semi-structured interviews

Two consented participants who did not complete the intervention (“non-engagers”) were interviewed. The interviews were audio-recorded to and transcribed. Interview topics included reasons for non-engagement and barriers to intervention engagement, opinions regarding the intervention, and feedback on potential modifications to the intervention (content, delivery, timeframe, etc.) to improve adherence and retention.

### Analysis

Based on the guidelines for pilot studies established by Leon et al., [40] the primary goal of the pilot study was to assess feasibility and acceptability of the study intervention and procedures. A target sample size of 15 was considered sufficient for completion of focus group procedures. Secondarily, we conducted preliminary efficacy analysis of the intervention through calculation of Cohen’s *d* effect sizes.

### Feasibility

Feasibility metrics for the study were informed by Leon et al. [40] and goals pre-determined in line with metrics that would indicate that the subsequent study would be feasible (recruitment/enrollment) and scientifically valid (intervention adherence, data availability, and intervention fidelity (Table 1).

**Table 1:** Pre-defined feasibility goals and outcomes

	Feasibility Goal
Screening	6 participants/month
Enrollment	50% of screened participants
Intervention Adherence	75% attendance
Fitbit wear	80% valid wear days
Retention	80% of PHQ-9 completed
Intervention Fidelity	≥ 3 on the QBAS-SF

### Acceptability

We conducted a descriptive analysis of data from the acceptability survey to assess particapnts’ perceived benefits of the intervention and opinions on the intervention delivery.

### Preliminary efficacy analysis

Though not designed or powered to conduct a formal efficacy analysis, we present results for PHQ-9, total steps, and MVPA (sum of “Very Active Minutes” + “Fairly Active Minutes”). We report raw data means at baseline and the final week of study participation and mean change from baseline to final week of study participation along with the Cohen’s *d* effect size (mean change/standard deviation of change). Total steps and MVPA outcomes were only considered valid for days with at least 600 min of wear time. Minutes of sleep and minutes with missing heart rate were excluded from wear time.

### Qualitative analysis

We developed a deductively-driven thematic codebook based on interview and focus group topics and a preliminary review of the data. We used a sample of the data (one interview and one focus group transcript) to modify and refine the codebook, adding codes for identified themes. The remainder of the data were double-coded. The coding team (a qualitatively trained research assistant and PC) met weekly to reconcile coding discrepancies and achieve consensus. We analyzed the data using an immersion-crystallization approach [41] to identify and characterize key themes and synthesize findings. Qualitative data were managed and analyzed using NVivo 12.0 (QSR International, Australia).

## Results

We screened 16 individuals and of these, 15 were eligible and enrolled in the study. One individual was ineligible due to a PHQ score ≤ 10. One participant withdrew from the study after completing 6 intervention visits due to ongoing chronic life stress and the need to seek a higher level of psychological care, but did complete post-intervention assessments at the time of withdrawal. Available data provided by that participant is included in the analyses reported below. Demographic characteristics of the enrolled participants are presented in Table 2.

**Table 2:** Demographic and clinical characteristics of enrolled participants

Female (%)	11/15 73.3%
Age (mean)	38.8 (SD = 11.7) Range: 23.1–64.4
Ethnicity	
Hispanic (%)	2/15 13.3%
Race	
White (%)	11/15 73.3%
Black (%)	2/15 13.3%
Asian (%)	2/15 13.3%
Marital Status	
Never Married	4/15 26.7%
Married	9/15 60.0%
Divorced	2/15 13.3%
Education	
Junior College or Technical School Diploma	4/15 26.7%
Some College	1/15 6.7%
Bachelor’s Degree	7/15 46.7%
Master’s Degree	3/15 20.0%
Employment	
Full Time	9/15 60.0%
Not Employed	2/15 13.3%
Retired	2/15 13.3%
Other	2/15 13.3%
Clinical Characteristics	
PHQ-9	16.8 (SD = 4.1) Range: 11–27
GAD-7	12.1 (SD = 5.2) Range: 2–21

### Feasibility

Feasibility data are presented in Table 3. All metrics exceeded the predetermined feasibility goal, with the exception of Fitbit wear and number of participants screened per month.

**Table 3:** Feasibility outcomes

	Feasibility Goal	Result
Screening	6 participants/month	3
Enrollment	50% of screened participants	93.75%
Intervention Adherence	75% attendance	96.67%
Fitbit wear	80% valid wear days	75%
Retention	80% of PHQ-9 completed	96.67%
Intervention Fidelity	≥ 3 on the QBAS-SF	4.21

### Acceptability

Acceptability of the intervention was evaluated through a post-intervention survey and focus groups. In the survey (Table 4), participants indicated that they believed the intervention helped them increase physical activity, with an average response of 4.20 (SD = 1.1) and decrease depressive symptoms with an average response of 4.33 (SD = 0.8) on a 5-point Likert scale (1-Strongly Disagree, 5-Strongly Agree). No serious or study-related adverse events were reported by participants.

**Table 4:** Acceptability Data

	Mean (SD)
Participating in these intervention sessions was important to me	4.93 (0.2)
The intervention helped increase my physical activity level	4.20 (1.1)
The intervention helped decrease my depressive symptoms	4.33 (0.8)
As a result of the intervention sessions, I believe I will be able to successfully increase my physical activity on my own	4.33 (1.0)
I am feeling better overall	4.33 (1.0)
My quality-of-life has improved	4.00 (1.2)
I enjoyed meeting with my therapist using the video technology	4.93 (0.2)
I experienced problems using the video technology during the intervention	2.53 (1.4)

### Preliminary efficacy

**Fig. 1:**
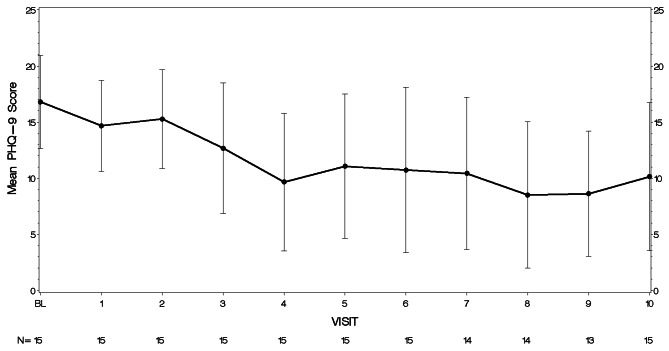
Mean PHQ-9 scores by visit

**Fig. 2:**
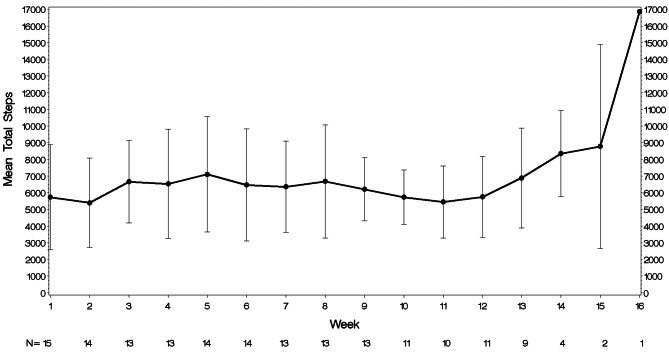
Mean number of steps by week

**Fig. 3:**
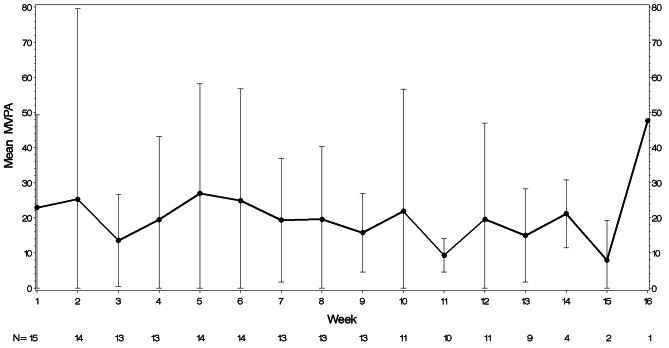
Mean MVPA by week

Preliminary efficacy of the intervention was also demonstrated, as the mean depressive symptom score on the PHQ-9 decreased from 16.8 (SD = 4.1), which is in the moderately severe range, at screening to 10.1 (SD = 6.6), in the moderate range, at Visit 10 resulting in a mean change of -6.7 (SD = 5.2, Cohen’s *d* = 1.3). Mean PHQ-9 scores by visit are shown in Fig. 1. Mean steps/day increased from 5729.3 (SD = 3143.6) at Week 1 to 6391.6 (SD = 3936.0) at the final week of study participation (mean duration of Fitbit use was 11.9 weeks (SD = 3.8)). The mean increase was 662.3 steps/day (SD = 4757.4, Cohen’s *d* = 0.14). Mean steps/day by week are shown in Fig. 2. Minutes MVPA as measured by the Fitbit decreased slightly from 22.9 (SD = 26.4) minutes/week at Week 1 to 20.7 (SD = 25.6) minutes/week during the final week of participation, a decrease of 2.1 min/week (SD = 33.5, Cohen’s *d* = 0.06). Mean minutes/week of MVPA are shown in Fig. 3. In contrast, self-reported minutes of MVPA from the PAVS increased from 22.00 (SD = 13.2) at baseline to 36.67 (SD = 20.6) in the final week of participation, a mean increase of 14.67 min (SD = 25.3, Cohen’s *d* = 0.58).

### Qualitative results

The final codebook consisted of seven thematic parent codes and three child codes, subcategories with detailed examples that were nested under the parent codes (Table 5). Using an iterative analysis process, we reviewed and summarized code reports, then grouped the codes into the following themes: (1) perceptions of the intervention; (2) participant experiences with tele-therapy; (3) intervention impact; and (4) suggestions for improvement. Two related themes, discussed below, were the convenience of tele-therapy and participants’ sense of accountability. These themes highlight the feasibility and acceptability of this intervention.

**Table 5:** Qualitative codes

Code	Definition	Example
Challenges	Barriers to engagement or completion of tele-therapy sessions	*“…keeping up with the apps and everything*,* I mean it requires effort*,* so I just felt like I shouldn’t commit.”* (PID1)
Engagement	Reasons for engagement and motivation to continue with intervention	*“I was in a very downward spiral and I realized that…something has to change…and I found this one and I was [like]*,* ‘Okay*,* this is a mild one*,* this is the first step to get better.”* (PID4)
Fitbit	Feedback about Fitbit device	*“It was my first fitness tracker…I’m a data geek so I really love having my exercise recorded and looking at my sleep patterns…”* (PID2)
Impact	Thoughts about the intervention’s impact	*“I had great weekly meetings with her [the therapist] and you know*,* I would say I gained a lot from that in general and try to pick up healthy habits.”* (PID8)
*Child codes under Impact*		
Physical activity	How the intervention did or did not affect participant’s level of physical activity.	*“I also found myself motivated to exercise*,* like several times I’ve finished the therapy session and then I would go exercise right afterwards…I found that enjoyable and helpful.”* (PID2)
Feelings and beliefs	Intervention impact on participants’ perceptions of themselves, quality of life, and mood, including depressive symptoms.	*“I thought it was…effective*,* kinda helping out with the moods and the conversations and walking through everything from emotions to events that happened that impacted the emotions.”* (PID6)
Expectations	Initial perceptions and expectations of the intervention	*“Starting off*,* I was a little worried that it was gonna be hard to hold myself accountable…I specifically wanted to make myself feel better and it turned out being very*,* very helpful.”* (PID4)
Non-engagers	Depressive symptoms, mental health, and physical activity non-engagers	*“You know*,* it’s [physical activity] kinda ebbed and flowed since I declined.”* (PID7)
Suggestions	Positive feedback on intervention components and suggestions for improvements	*“If it [the intervention] was three or four months*,* it would really be more helpful in getting a pattern set for me.”* (PID12)
*Child codes under Suggestions*		
Complaints	Negative feedback on intervention components.	*“…when it came to the paperwork*,* that’s where I was like ugh man…so that was probably my dislike about the whole study.”* (PID10)

### Initial perceptions of the intervention

Overall, participants had positive perceptions of the intervention. They cited several reasons for why the study appealed to them: (1) the convenience of tele-therapy (2), the combination of therapy/emotional support and physical activity (3), a personal desire to increase their physical activity and/or get into shape, and (4) a recognition for change. As one participant (Participant 8) noted, “*I think like just having that weekly meeting with a therapist and combining therapy with physical activity*,* yeah that’s- that’s 100% what appealed to me*.” While non-engagers also had positive perceptions of the intervention, they declined to participate because they felt they could not commit the time and effort required by the study. One non-engager (Participant 7) noted, “*I felt like it seemed like a good study. I just wasn’t at the time like committed to fully doing everything that the study needed and…I was just like well if I’m worried about the time commitment…I’m gonna have to pass for now*.” The other non-engager (Participant 11) shared, *“…keeping up with the apps and everything I mean it requires effort…I felt that if I wasn’t gonna to be able to commit then that’s why I pulled out.*”

### Tele-therapy

Overall, completers did not have issues using the virtual platform for tele-therapy. Only one completer experienced technical issues, but the therapist worked with them to resolve connectivity issues. Among the completers, there was universal support for the use of a virtual platform. Completers liked the convenience and flexibility of tele-therapy. One participant (Participant 2) stated, “*I thought it was very convenient*,* you know*,* being able to do it from home or work or anywhere*,* so*,* I really liked that aspect of it.*”

Some completers particularly appreciated the convenience of tele-therapy, comparing it to their previous experiences with in-person therapy which required them to rearrange work schedules and find accommodations for childcare. One completer (Participant 4) remarked, “*I found it more convenient for me because with my schedule just to go to an appointment*,* long enough to go to the appointment*,* stop see the therapist*,* come back*,* rearrange my schedule with my children*,* it just was easier to pop in*,* pop out*.” Others had also become accustomed to using virtual platforms because of remote work, Participant 9 shared “*For me*,* part of it was the telehealth…now working remote and just doing everything from home*,* it just added to that convenience*,* and I’ve had to drive to the therapist office before and you have to take out two to three hours out of your day*,* you know*,* drive time*,* sitting there*,* driving back*,* so you’re really taking off a lot of time from work and this way it’s just a quick*,* you can…get it done. So that was the additional component for me*,* just the convenience of the tele-therapy.*”

One completer (Participant 11) expressed initial unease about tele-therapy, noting they were afraid it would blur the boundaries between home and therapy. They joined the study despite their reluctance and acknowledged the virtual platform made it easier for them to attend sessions. They shared, “*…in my experience it’s easier to go to an office and walk away from it rather than having those moments at home where it’s kinda like my personal space*,* but…I think that if I actually had to go to an office*,* I may not have put in the effort. I may not have met up with her for each of our sessions*,* it [tele-therapy] did make it easier*,* and I didn’t have the struggle that I felt that I would doing the sessions at home. So*,* I went into it very*,* very hesitant and almost backed out of it*,* and I was glad that I didn’t. It was better than I thought*.” The convenience and accessibility of tele-therapy spurred their consistent attendance, providing a sense of accountability. Accountability was another theme in focus groups when discussing the intervention’s impact.

### Intervention impact

Completers had positive feedback about the intervention, noting its impact on their mood, quality of life, and perceptions of themselves. They had favorable experiences with tele-therapy sessions and particularly appreciated talking to the therapist about their thoughts and feelings. They described how the therapist helped them process emotions, recognize triggers, and develop and implement new positive behaviors. One participant (Participant 5) shared, “*I went through a lot also during these past few weeks*,* a whole lot of personal stuff and being able to have someone to talk through it with was really helpful and that I was able to process my emotions*,* get through it and get up and do the things that I knew I needed to do but didn’t want to do*.” Another completer (Participant 3) echoed this sentiment, “*…so she [the therapist] was really helping me more with the mindset I would say. It was extremely helpful…I had quite a few kind of major life events that happened during that time*,* so she really helped me kind of navigate through some of my thought processes*.” This individual elaborated, “*I’m definitely recognizing more what triggers me or when I’m having those moments when I’m about to go down a path that’s not good so…I’m able to recognize when that inner critic in my brain…and the techniques and the ways to stop it*,* it definitely has been a game changer so I don’t stay on those low parts or low points as long as I did before because now I can see that there’s a pretty easy way out and I’m okay and I need to give myself permission to get myself out of there*.”

Completers also noted how the therapist helped reframe their perspectives, including their self-perceptions. They described learning to prioritize themselves and being kinder to themselves. For example, one completer (Participant 2) shared how therapy reframed their perspective about experiencing depressive symptoms, noting “*…as far as like my depression symptoms at the beginning of the study versus the end*,* you know*,* she [the therapist] really helped me to see*,* to be a little easier on myself and not beat myself up for being human and having off days*,* you know*,* so that was really encouraging to do that*.” Another completer (Participant 8) expressed similar feelings about how therapy taught them to be less harsh on himself, “*I found tele-therapy likewise extremely beneficial…like one of the things that we honed in on was me not being so hard on myself. I can beat myself up quite a bit about even the most minor things so…we talked a lot about sort of the metaphorical punching bag that I like to hit because I enjoy boxing and how I kinda beat myself up a lot and it helped me a lot to forgive myself and to take it [a little easy]*,* yeah.*”

While the intervention positively affected completers’ mood and perceptions of themselves, its impact on their level of physical activity varied. Some completers increased their physical activity while others remained sedentary and unmotivated. For example, one completer said the weekly tele-therapy sessions were helpful for their depression, but also did not become more physically active because of their depression. Conversely, another completer (Participant 9) had become accustomed to staying home and intervention helped them develop a routine to become more active which reduced their depressive symptoms, noting, “*I was just at home. Like I would work from home and stay home and not do anything…. but now I’m much more active. I’ll go to the gym three*,* four times a week for 20 or 30 minutes…it’s created a routine for me that has helped me just with my depression and the way that I think and feel about myself*.” Another completer (Participant 2) explained how the tele-therapy sessions motivated them to exercise and provided a sense of accountability, “*I really liked the therapy sessions cause we were able to talk through a lot of my ups and downs and what was happening for me and I also found myself motivated to exercise*,* like several times I’ve finished the therapy session and then I would go exercise right afterwards and then it being week to week*,* it kept me accountable too*,* like okay I need to exercise cause my appointment’s coming up so that helped. I found that enjoyable and helpful*.”

Although completers had mixed physical activity results, there was consensus about how the therapist helped them view exercise differently – as something positive. As they discussed how the therapist helped reframe their perspectives about themselves, they noted this reframing of perspectives also applied to their view of physical activity. One completer (Participant 2) learned to connect exercise and their mood, “*The study helped to link my emotional state and my moods to exercise to where before I was – I don’t really enjoy exercise…and then the study helped me to get a little bit more into a rhythm*,* but then also to see the benefits on more than just a physical level*.” Another completer (Participant 4) shared similar feelings, “*It helped me understand the feelings of why the exercise was helping me and helping me understand okay*,* you don’t have to do all the things to make everybody else better that you could take time for yourself and it’s okay to choose something that is for you*,* even if it’s just the exercise. You don’t have to benefit anybody else*,* it’s to benefit you. She helped me understand that…*”.

### Participant suggestions for improvement

Participants offered several ideas to improve the intervention. The two most frequently mentioned suggestions were an electronic format to record their information and more structure for the physical activity component of the intervention. Completers wanted an electronic format to record and track their activity and moods instead of a paper notebook. For example, one participant suggested integrating Fitbit and self-recorded survey data, noting discrepancies between the two data sources. This suggestion may only work for participants who regularly wore and synced their Fitbit devices. Some participants experienced issues with the Fitbit, such as failing to wear and/or sync the device, inaccurate tracking, and broken wristbands. Completers also suggested increasing the frequency of logging their information rather than once a week per study design. One completer (Participant 3) shared, “*The only thing I would change… you’re keeping track of that information [electronically] versus having like a paper notebook or…having something where you could just do it daily…it felt very heavy at the end of the week to have to enter all of your physical activity for the entire week.*” Completers indicated they preferred filling out information about their mood and physical activity in the moment or on a daily basis because they struggled to recall activities from previous days. The other suggestion was a request for more structure for physical activity requirements, such an exercise schedule or mini-goals, to provide added accountability. While participants appreciated the flexibility, some desired structure. One completer (Participant 8) shared, “*I think having some sort of set schedule rather than it being a little more loosey-goosey because I didn’t increase my physical activity during the study… the tele-therapy was very beneficial in so many other ways*,* but I didn’t feel like the accountability was there. Like I just didn’t feel like I was really meeting my goals as far as physical activity was concerned at all.*” Completers also listed other suggestions, such as providing a gym membership, having a peer accountability buddy, and increasing the length of the intervention to allow sufficient time for participants to establish and sustain good habits.

## Discussion

The primary purpose of this pilot study was to evaluate the feasibility and acceptability of the adapted BA intervention and study procedures. Results suggest the adapted approach to BA teletherapy is acceptable to participants. In both survey responses and focus groups, participants reported overall satisfaction with the intervention. In particular, participants noted the convenience and flexibility of delivering the intervention via teletherapy. Participants also reported perceived benefits from the intervention in the post-intervention survey, which were reinforced in the focus groups. These perceived benefits were further supported by our preliminary data demonstrating a significant reduction in depressive symptoms following the 14-week intervention.

The feasibility data reported from this pilot study largely supports the conduct of larger trials to evaluate the efficacy of the intervention. Pre-defined metrics for enrollment, intervention adherence, and intervention fidelity were exceeded in the pilot data. The screening volume metric did not meet the pre-defined criteria; however, this is a reflection of the higher than anticipated screening success rate (93.75% compared to an anticipated rate of 50%) rather than difficulty in participant recruitment, meaning that fewer participants had to be screened each month to meet recruitment goals.

Our evaluation of the preliminary efficacy of the intervention demonstrated a slight reduction MVPA as measured by Fitbit though an increase in steps per day was observed. Conversely, self-reported minutes of MVPA increased from Week 1 to the end of study participation. We note that given the small sample size, we did not conduct significance testing and that these preliminary results should be interpreted with caution. The discrepancy between self-reported and objective measures might reflect a desirability bias in participants’ responses to the self-report survey. Alternatively, the discrepancy could reflect a potential “compensation” effects, in which a reduction in incidental physical activity coincides with beginning a more structured physical activity program [42]. In our study, compensation effects could have occurred resulting in a reduction in incidental (or “non-exercise”) physical activity that is likely to be captured by objective measurement but not by self-report and intentional physical activity (“exercise”) more likely to be reflected in self-report. While we report both objective and self-report physical activity, we note that an objective measure of physical activity will serve as the primary physical activity outcome in future studies.

Previous research studies have examined combining BA with either structured exercise programs or interventions that aim to increase physical activity [27–30]. These studies have often reported intervention adherence rates of 50% or lower. In contrast, particapnts in our study attended 97% of intervention visits. The improved adherence rate observed in our trial may be the result of the briefer intervention. For example, the intervention delivered by Schneider et al. [27] lasted for 16 weeks and involved 38 intervention sessions. Another source of improved intervention adherence could be attributed to the teletherapy format for intervention delivery. Szuhany et al. [30] conducted a trial in which BA plus a physical activity intervention was delivered with 9 intervention sessions delivered over a 12-week period. While the intervention duration and number of intervention sessions was similar to our intervention, the intervention by Szuhany et al. was delivered in-person and resulted in 77% adherence and 40% of randomized particapnts did not complete the follow-up data collection visit. In another study [29], a self-guided web-based BA plus physical activity also resulted in poor adherence, with the median completion of 3 modules out of an available 8 weekly modules, suggesting the lack of interpersonal interactions might hinder adherence.

The results of the pilot study also suggest potential avenues for future research in this area. This study was part of a phased pilot project [26], and we implemented changes in the study protocol to improve the participant experience and ensure data availability (i.e., Fitbit troubleshooting and reminders) prior to the start of a larger pilot phase. However, some suggestions made by participants were beyond the scope and logistical constraints of the subsequent pilot but could inform future studies. The outcome data and qualitative feedback suggests variability in changes in physical activity across participants. In the focus groups, participants noted a need for additional intervention support strategies such as access to exercise facilities and social support. These findings indicate the potential for a multicomponent intervention or an adaptive intervention that provides additional intervention strategies for those who do not increase physical activity in response to the base intervention. For example, the use of an electronic or “mobile” workbook for tracking activity and goal completion could be examined as a means for improving intervention adherence and efficacy and could be evaluated in a trial in which participants are randomized to either the standard intervention or the intervention plus electronic workbook. Another aspect for future exploration is providing a more structured physical activity “prescription”. While our current intervention is intended to provide flexibility in completing physical activity, at least one participant indicated a desire for more structure. Future iterations of our intervention manual could incorporate increased assessment and collaboration in situations where some participants would prefer a more structured approach to their physical activity. Furthermore, while our aim is to provide flexibility in physical activity completion, a more structured approach for physical activity could be implemented in which “target” goals (i.e., minutes of weekly physical activity) are provided to participants.

Strengths of the study include use of both quantitative and qualitative data in the evaluation of feasibility and acceptability, the manualized approach to intervention delivery, and objective measurement of physical activity. While the current study seeks to gather participant feedback through surveys and qualitative research, we note that members of the target population were not involved in the initial design of the study or the intervention. Future efforts that involve member of the target population in the planning phase of the intervention and study development could yield valuable insights to improve the feasibility and acceptability of future studies. The small sample size of the current study, while reasonable for this stage of the research process and sufficient to provide acceptability data, limits our ability to evaluate the efficacy of the intervention on physical activity and depression outcomes. As such, we reported mean changes and Cohen’s *d* effect sizes observed in the study but chose not to conduct formal statistical test. Similarly, the single-arm design also limits evaluation of intervention efficacy. Subsequent randomized controlled trials would be necessary to determine efficacy and effectiveness of the intervention on physical activity and depression outcomes.

## Conclusions

While the preliminary data suggests the intervention can reduce depressive symptoms and potentially increase physical activity, these data should be interpreted cautiously given the small sample size and lack of a comparison group. Larger more rigorous trials are necessary to evaluate efficacy of the intervention on both depressive symptoms and physical activity. The acceptability and feasibility data from this pilot study suggests such a trial can be implemented successfully.

## Electronic supplementary material

Below is the link to the electronic supplementary material.

Supplementary Material 1

Supplementary Material 2

Supplementary Material 3

## Data Availability

No datasets were generated or analysed during the current study.

## Abbreviations

MVPA: Moderate-to-vigorous physical activity
PAVS: Physical Activity Vital Sign
BA: Behavioral Activation
PHQ: Patient Health Questionnaire
QBAS-SF: Quality of Behavioral Activation Scale - Short Form
