# Supplementary material for: Pilot evaluation on an adapted tele-behavioral activation to increase physical activity in persons with depression: a single-arm pilot study
Source: BMC Psychol. 2024 Nov 9;12:643. doi: 10.1186/s40359-024-02053-5 (PMC11549759; doi:10.1186/s40359-024-02053-5)
Supplement: Supplementary file 2 — Supplementary Material 2 [file 40359_2024_2053_MOESM2_ESM.docx]

Patient ID: ___________ Date: ______________

Participating in these intervention sessions was important to me

1 2 3 4 5

Disagree Disagree Somewhat Neutral Agree Somewhat Agree

The intervention helped increase my physical activity level

1 2 3 4 5

Disagree Disagree Somewhat Neutral Agree Somewhat Agree

The intervention helped decrease my depressive symptoms

1 2 3 4 5

Disagree Disagree Somewhat Neutral Agree Somewhat Agree

As a result of the intervention sessions, I believe I will be able to successfully increase my physical activity on my own

1 2 3 4 5

Disagree Disagree Somewhat Neutral Agree Somewhat Agree

I am feeling better overall

1 2 3 4 5

Disagree Disagree Somewhat Neutral Agree Somewhat Agree

My quality-of-life has improved

1 2 3 4 5

Disagree Disagree Somewhat Neutral Agree Somewhat Agree

I enjoyed meeting with my therapist using the video technology

1 2 3 4 5

Disagree Disagree Somewhat Neutral Agree Somewhat Agree

I experienced problems using the video technology during the intervention

1 2 3 4 5

Disagree Disagree Somewhat Neutral Agree Somewhat Agree

The technology issues I experienced reduced the quality of my sessions

1 2 3 4 5 6

Disagree Disagree Somewhat Neutral Agree Somewhat Agree Did not experience any problems

The number of therapy sessions was

1 2 3

Too few Just right Too many

The time between therapy sessions was

1 2 3

Too short Just right Too long

What has been the most helpful part of the intervention?

___________________________________________________________________________________

What has been the thing that frustrated you most about the intervention?

___________________________________________________________________________________

What else could the intervention have done to help you increase your physical activity?

___________________________________________________________________________________

What barriers made it challenging to complete the intervention?

___________________________________________________________________________________

What would help to reduce such barriers for future participants?

___________________________________________________________________________________

In the future, I believe this intervention could be completed by the following kinds of interventionists: [check all that apply]

- Someone without mental health experience or training
- A person with basic mental health training (like a graduate or medical student)
- A person with intermediate mental health training (like a licensed Social Worker)
- A person with advanced mental health training (like a licensed Psychologist or Psychiatrist)

In the future, I believe this intervention could best be done in the following kinds of settings: [check all that apply]

- Remote/from home using technology
- In-person at a research facility (like at UT Southwestern Medical Center)
- In-person at a doctor’s office (like your primary care physician)
- In-person at a recreation or fitness center

If given the choice, I would have preferred to complete the intervention:

- From home or a remote setting, via telephone
- From home or a remote setting, via live video conferencing
- In person

Do you have any other feedback you’d like us to know?

___________________________________________________________________________________

___________________________________________________________________________________

**Thank you for participating in our study!**
