# Supplementary material for: Pilot evaluation on an adapted tele-behavioral activation to increase physical activity in persons with depression: a single-arm pilot study
Source: BMC Psychol. 2024 Nov 9;12:643. doi: 10.1186/s40359-024-02053-5 (PMC11549759; doi:10.1186/s40359-024-02053-5)
Supplement: Supplementary file 3 — Supplementary Material 3 [file 40359_2024_2053_MOESM3_ESM.docx]

**Initial Session Questionnaire**

Patient ID: _______ Date: ______________

Are you glad that you came to this intervention session?

YES NO UNDECIDED

Do you plan to return to the next intervention session?

YES NO UNDECIDED

What is one thing you liked about this intervention session? ______________________________________________________________________________

______________________________________________________________________________

What is one thing you disliked about this intervention session? ______________________________________________________________________________

______________________________________________________________________________

What is one thing that might make it difficult for you to attend the intervention each week?

______________________________________________________________________________

______________________________________________________________________________

What is one thing that might make it easier for you to make it to the intervention every week?

______________________________________________________________________________

______________________________________________________________________________

What is one thing you learned from this intervention session that will help you be more physically active?

______________________________________________________________________________

______________________________________________________________________________
